# Disrupting Osr1 expression promoted hepatic steatosis and inflammation induced by high-fat diet in the mouse model

**DOI:** 10.1371/journal.pone.0268344

**Published:** 2022-06-03

**Authors:** Ernest C. Lynch, Zhimin Liu, Lin Liu, Xian Wang, Ke K. Zhang, Linglin Xie

**Affiliations:** 1 Department of Nutrition, Texas A&M University, College Station, TX, United States of America; 2 Center for Epigenetics & Disease Prevention, Institute of Biosciences & Technology, College of Medicine, Texas A&M University, Houston, TX, United States of America; Universite du Quebec a Montreal, CANADA

## Abstract

NAFLD, regarded as the hepatic manifestation of metabolic syndrome, is the most common form of liver disease in the United States. The Odd-skipped related 1 (Osr1) gene was previously reported to play a critical role in embryonic development and as a cancer repressor gene, however its role in overnutrition induced fatty liver disease has never been explored. Induced by a high-fat diet (HFD) for 10-week, the development and the progression of NAFLD was evaluated in either Osr1 heterozygote (Osr1 group) or wildtype mice (WT group). The Osr1 mice, regardless of sex, exhibited more severe steatosis compared to WT. Upregulation of lipogenesis protein including Srebp1c was detected in the Osr1 group, together with impaired IRS2 expression and overactivated Akt/mTOR signaling. In addition, the Osr1 mice had decreased bile acid synthesis in the liver with depressed hepatic expression of *Cyp7a1* and *Cyp27a1*. Furthermore, there was more macrophage infiltration with enhanced expression of *Il-1β* and *TNF-α* in the Osr1 liver, associated with overactivation of JNK and NF-κB signaling. In summary, our study showed that Osr1 plays an important role in regulating the lipid homeostasis and hepatic inflammation, whose disruption contributes to NAFLD progression.

## Introduction

Nonalcoholic fatty liver disease (NAFLD), widely considered as the hepatic manifestation of metabolic syndrome, [1] is the most common cause of chronic liver disease in the United States, affecting an estimated 64 million adults with a current annual medical cost of about $103 billion [1]. Further, 25% of the global adult population is affected by NAFLD.(2) With the prevalence and severity of obesity increasing at an alarming rate, the prevalence of all classifications of NAFLD is expected to increase [2]. Nonalcoholic fatty liver disease encompasses a comprehensive clinical spectrum of conditions, ranging from simple steatosis, to nonalcoholic steatohepatitis (NASH), fibrosis, cirrhosis, and hepatocellular carcinoma. The more severe circumstances of this spectrum can lead to end-stage liver failure and death.

Insulin resistance resulting in hyperinsulinemia, is the primary pathogenic mechanism in NAFLD [3]. This leads to an overabundance of serum free fatty acids from continuous lipolysis of visceral adipose tissue. These free fatty acids are then translocated to the liver, where they are reassembled into triglycerides, serving as the predominant source of hepatic lipids in NAFLD. Other sources of hepatic triglycerides include de novo lipogenesis (DNL) and dietary fat intake [4]. Steatosis occurs when intrahepatic fat accumulates to 5% of total organ weight. These lipids inundate mitochondrial oxidative abilities, and reactive oxygen species (ROS) are produced, activating the metabolic cascade that promotes NASH from simple steatosis. The accumulation of lipids in hepatic tissue served as the inceptive insult in the development of NAFLD, and originally conceptualized in the “two hit” pathogenesis theory [5] until recently when a more accepted “multiple parallel hit” model was proposed [6]. New consideration is given to environmental factors, genetic and epigenetic influence, and variation in crosstalk between various tissues and organs including adipose tissue, gastrointestinal tract, pancreas, and the liver to provide a more accurate explanation [6]. A multitude of potential sources of injury have been presented, including mitochondrial ATP dysfunction, systemic hypoxia, dysregulated adipokine production, high carbohydrate diets, depleted mitochondrial glutathione, and paradoxically rapid weight loss among others [7–10].

The odd-skipped related 1 (Osr1) gene encodes a putative transcription factor, a 266 amino acid protein containing four Cys2His2-type zinc finger domains [11]. The homolog Odd-skipped related 1 (Odd1), first discovered in drosophila, is responsible for modulating several genes required for correct segment formation in developing embryos [12, 13]. In murine models, Osr1 was found to play a profound role in kidney, limb, urogenital, and cranio-facial development [14–18]. In addition, Osr1 exerts an anti-proliferative effect, inducing cell cycle arrest and apoptosis in multiple cancer cell lines [15, 19].

To investigate the potential role of Osr1 in the progression of hepatocellular carcinoma (HCC), our lab adopted a mouse model using hepatotoxic and carcinogenic Diethylnitrosamine (DEN) and high-fat diet (HFD) treatment for 8 weeks [20]. We have shown that Osr1 heterozygous mice displayed more severe NASH with higher serum alanine aminotransferase (ALT) levels than the WT mice. The Osr1 heterozygous mice also revealed early signs of liver fibrosis with more hepatic inflammation [20]. With this novel discovery, we hypothesize that Osr1 also plays a protective role in the progression of NAFLD induced by an obesogenic diet. Using the NAFLD model induced by 60% HFD for 10 weeks, liver pathophysiology focusing on hepatic steatosis and inflammation and their associated signaling pathways are examined in the Osr1 heterozygous and wildtype (WT) mice.

## Materials and methods

### Study design

The Osr1 heterozygote (*Osr1*^*+/-*^) mice were purchased from the Jackson Laboratory (Strain# 009387, B6.129S1-*Osr1*^*tm1Jian*^/J). Three-week-old Osr1 heterozygote mice and their littermate control mice (C57/BL6) were fed a high fat diet (60% kcal from fat, HF) for 10 weeks (n = 9 for male and n = 5 for female mice). Mice were housed in a specific pathogen-free (SPF) room set at 22°C ± 2°C, 55% ± 5% humidity, and 12 hour/12 hour circadian rhythm. Food and water were provided *ad libitum*. The mice were humanely sacrificed by CO_2_ inhalation for a period spanning over 2 minutes and were subjected to cervical dislocation to ensure death. Mouse experiments were completed according to a protocol reviewed and approved by the Institutional Animal Care and Use Committee of Texas A&M University, in compliance with the USA Public Health Service Policy on Humane Care and Use of Laboratory Animals.

### Diet composition

Diet was purchased from Research Diets, LLC (New Brunswick, NJ) to implement a diet-induced obesity (DIO) model. The HF diet (Cat#D12492) had an energy density of 5.24 kcal/g (60% fat energy, 20% carbohydrate energy, and 20% protein energy). The fat source is composed of 54% lard and 6% soybean oil.

### Antibodies

Antibodies against mTOR, phospho-mTOR, IRS2, AKT, phospho-AKT-308, NF-kappaB, phospho-NF-kappaB, JNK, phospho-SAPK/JNK (Thr183/Tyr185), PPAR-g, F4/80, SREBP, and GAPDH were purchased from Cell Signaling Technology (USA).

### Intraperitoneal glucose tolerance test (IPGTT)

To test glucose tolerance, an IPGTT was performed prior to termination at week 10. Mice were fasted overnight and injected intraperitoneally with a glucose solution in saline the following morning. Glucose solution consisted of 20% D-glucose in water with the administration dose of 2.0 g/kg body weight. Plasma glucose levels (mg/dL) were measured from tail blood by a handheld blood glucose meter (Contour next EZ, Ascensia Diabetes Care US, Inc., Parsippany, NJ, USA) before (blank) and at 15, 30, 60, and 120 minutes after glucose injection. Area under curve (AUC) analysis was used to assess glucose tolerance. Glucose solution was purchased from Sigma-Aldrich (USA).

### Serum triglyceride (TG) measurement

Serum triglycerides (TG) were detected using a triglyceride calorimetric assay (Cayman Chemical Company, Ann Arbor, MI, USA) according to the manufacturer’s instructions.

### Bile acid synthesis assay

Physiological concentration of total bile acids (TBA) in liver tissue were assessed using a total bile acids assay kit (calorimetric) (Abcam Biotechnology, Cambridge, MA, USA) according to manufacturer’s instructions. For bile acid extraction, 100 mg of liver tissue was homogenized in 95% molecular grade ethanol and incubated overnight in a 60°C water bath. After the sample was centrifuged at 3500 RPM for 10 minutes, the supernatant was carefully collected, transferred to a 2 mL microtube, and stored at -80°C. The tissue pellet was resuspended and vortexed in 80% molecular grade ethanol, followed by an overnight incubation in 60°C water bath. After another centrifugation at 3500 RPM for 10 minutes, the supernatant was combined and returned to -80°C storage. Tissue pellets were again resuspended and vortexed in a 2:1 (v:v) molecular grade chloroform: methanol. Samples were returned to 60°C water bath for overnight incubation. Total supernatant was extracted and combined after samples were centrifuged at 3500 RPM for 10 minutes. 5 μL supernatant for all samples were used to test for total bile acids, using 80% molecular grade ethanol as a blank.

### Realtime-PCR (RT-PCR)

Total RNA was extracted using TRIzol reagent (Thermo Fisher Scientific, USA), and the concentration was determined in triplicate using a NanoDrop ND-1000 spectrophotometer (Thermo Scientific). Total mRNA (1ug) was amplified and reverse transcribed using ReadyScript®cDNA Synthesis Mix (Sigma-Aldrich, USA), and RT-PCR was performed using BioRad SYBR Green Master Mix on a CFX384^TM^ Real-Time System (BIO-RAD, USA) with the CFX Manager 3.1software. Primers used for RT-PCR analysis are shown in Table 1.

**Table 1:** Primer used for RT-PCR analysis.

Gene	Forward sequence (5′- 3’)	Reverse sequence (5′- 3’)
*ACC*	ATCCAGGCCATGTTGAGACG	AGATGTGCTGGGTCATGTGG
*Cd36*	TGGAGGCATTCTCATGCC-AG	TTGCTGCTGTTCTTTGCC-AC
*cyp27a1*	TTGCCTGGATAGGGCTCATAG	GTGGGGCACTAGCCAGATTC
*Cyp4a10*	CAAAATCCAAGGCCTGAACATCA	ATGGAGAAACTCGTGTGAGGATT
*Cyp4a12b*	GAGTGTCCTCTAATGGCTGCTTG	CCACTTCAGCACGAAGGTCC
*Cyp4a14*	ATAGGAACAGCTTGTCTGATGAGG	CCCAATGCAGTTCCTTGATCC
*Cyp4a31*	CCCAAGTGCCTTTCCTCGAT	AAACCATACCCTGATCGCCC
*Cyp7a1*	CTGGGGGATTGCTGTGGT-AG	GCACAGCCCAGGTATGGA-AT
*Ehhadh*	TTGGACCATACGGTTAGA-GCC	CACTGGCTTCTGGTATCG-CT
*Fabp*	GTGGTCCGCAATGAGTTC-AC	GCTTGACGACTGCCTTGA-CT
*FAS*	TGCTTGCTGGCTCACAGTTA	GCTTGACGACTGCCTTGA-CT
*IL10*	GCTCTTACTGACTGGCATGAG	CGCAGCTCTAGGAGCATGTG
*IL1b*	GCAACTGTTCCTGAACTCAACT	ATCTTTTGGGGTCCGTCAACT
*IL4*	GGTCTCAACCCCCAGCTAGT	GCCGATGATCTCTCTCAAGTGAT
*IL6*	GCCTTCTTGGGACTGATGCT	TGTGACTCCAGCTTATCTCTTGG
*plin4*	TCTGAACAGACAGCTGGAGA	CAGTCCACCCTGGACCATTC
*Plin5*	GGATACACGCGGGATCTGAG	CCACCACTCGATTCACCACA
*Ppary*	TGGTTCAAATATGCCACC-AG	CCAAGTGCTGGGATTAAA-GG
*SREBP-1c*	AGCAGTCACCAGCTTCAG-TC	GGTCATGTTGGAAACCAC-GC
*TNFa*	ACCCTCACACTCACAAACCA	ATAGCAAATCGGCTGACGGT

### Statistical analysis

All data was analyzed through statistical analysis. For longitudinal data including body weight, a linear mixed model was used for the analysis of repeated measures. For others, one-way ANOVA were carried out by using GraphPad Prism 7 software (GraphPad Software, San Diego, CA, USA).

## Results

### The Osr1 heterozygous female mouse gained more body weight induced by the HF diet

**Fig 1:**
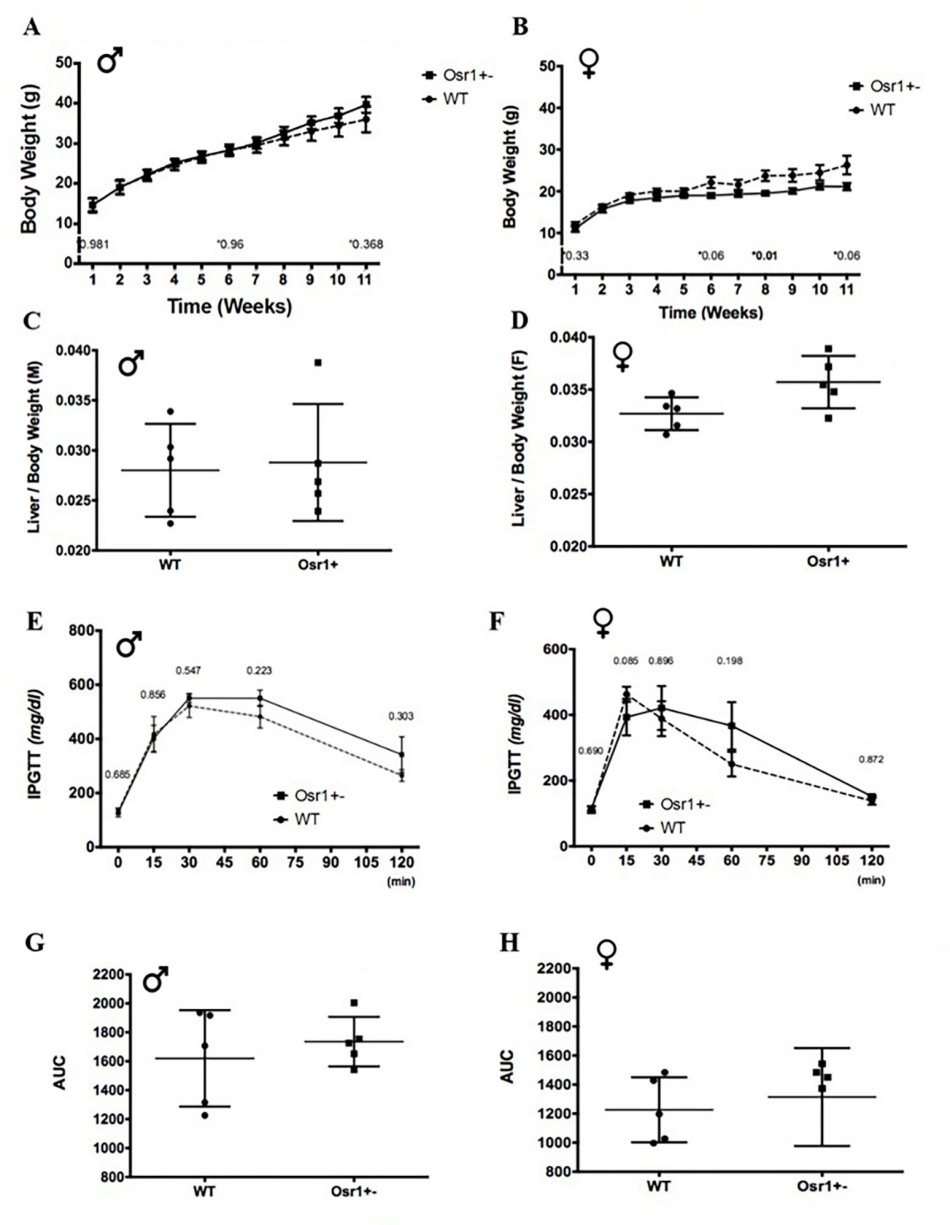
Body weight and glucose tolerance during the experimental period. (A). Male mice body weight growth log from weaning to week 10 indicated no significant difference between groups. (B). Female Osr1 mice started to gain more body weight at week 6. (C). Male mice organ to body weight ratio exhibited no significant difference between groups. (D). *Osr1* heterozygous female mice showed larger liver weights. (E). At week 10, no significant differences were noted in glucose tolerance between male mice groups. (F). Male mice IPGTT area under curve. (G). Week 10 IPGTT indicated no significant difference between female mice groups. (H). Female mice IPGTT area under curve. AUC: Area Under Curve. Data is presented as Mean±SD, N = 5–7; *p<0.05.

Compared to the WT group, the Osr1 group was weaned at a similar body weight regardless of sex. There was no significant difference in body weight between WT and Osr1 male heterozygous mice at each experimental week point (Fig 1A). At sacrifice, there was no statistical difference in liver to body weight ratios between WT and Osr1 mice observed in males (Fig 1C). However, female Osr1 mice started to show a heavier body weight at week 6 and the difference in body weight maintained until being sacrificed (Fig 1B). The Osr1 female mice sustained marginally higher liver weights by comparison when it was normalized against total murine body weight (P = 0.05, Fig 1D).

Glucose tolerance was measured by performing an IPGTT before termination at week 10. Fasting glucose was not significantly different between groups (Fig 1E and 1G). All mice regardless sex returned to basal level at 120 minutes. Confirmed by AUC measurement, the WT and Osr1 mice in general responded similarly to glucose challenge (Fig 1F and 1H).

### Osr1 heterozygous mice exhibit more severe hepatic steatosis induced by the HF diet

**Fig 2:**
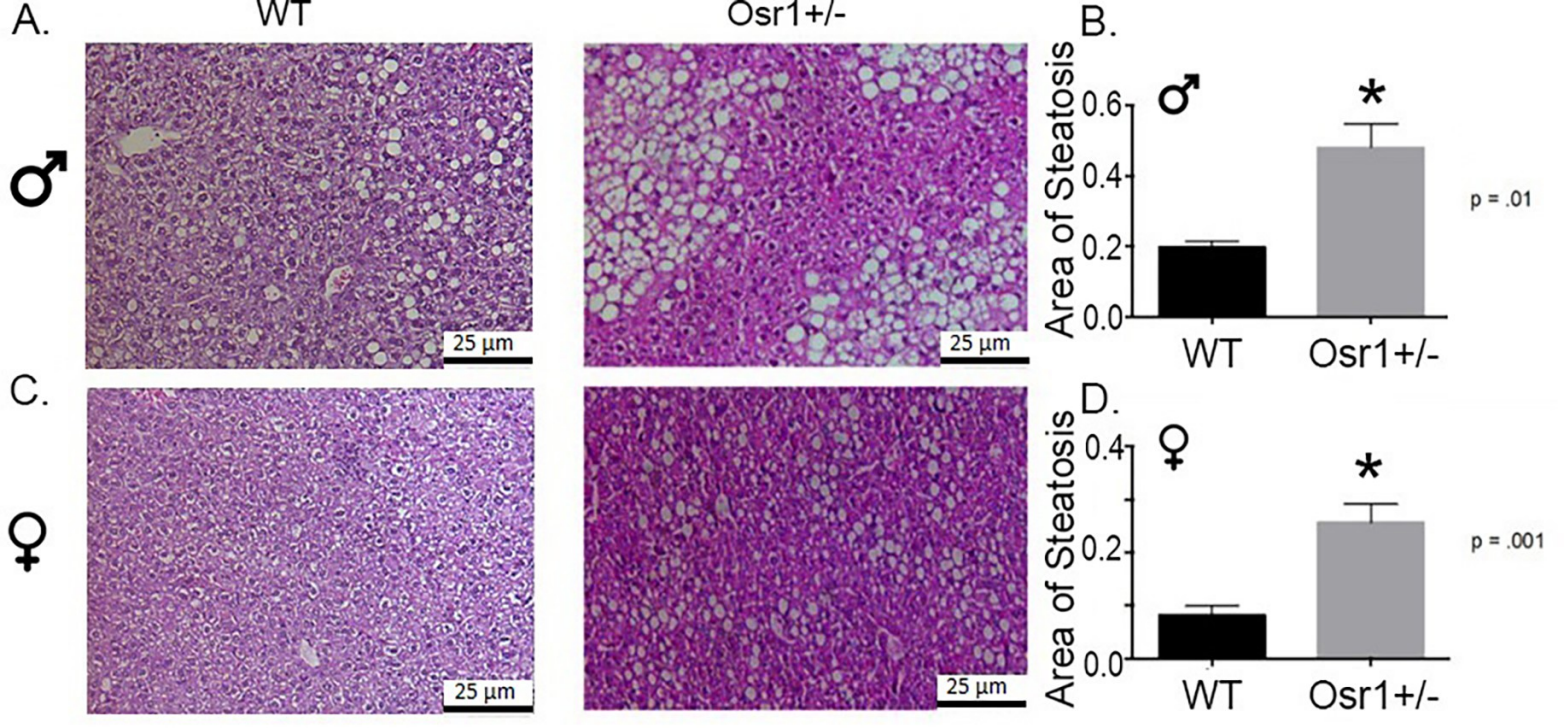
Osr1 heterozygous mice exhibit more severe hepatic steatosis induced by the HF diet. (A). HE staining of male liver tissue. (B). *Osr1* heterozygous male mice had significantly larger area of steatosis. (C). HE staining of female liver tissue. (D). *Osr1* heterozygous female mice had significantly larger area of steatosis. Scale = 25 μm. Data is presented as Mean±SD, N = 5; *p<0.05.

Hepatic histology was examined to evaluate if Osr1 downregulation influenced the severity of NAFLD. When compared to the WT group, Osr1 heterozygous mice had significantly more steatosis. WT male mice displayed little or mild microvesicular steatosis, with most lipid deposition observed as minor intracytoplasmic vacuoles. Osr1 mice however showed mild to severe hepatosteatosis, with clustered and ballooning perivenular lipid macrovesicles (Fig 2A). When comparing the total area of steatosis between male mouse groups, Osr1 heterozygous mice presented with statistically greater (p<0.01) lipid accumulation (Fig 2B). Female WT mice exhibited sparsely dispersed lipid microvesicles, and no macrovesicular steatosis to any observable degree (Fig 2C). Hepatocytes maintained centrally located nuclei in WT females. By comparison, Osr1 female mice had a far more severe phenotype of fatty liver. Similar to heterozygous males, Osr1 females displayed profound lipid macrovesicles (Fig 2C). The more severe hepatic steatosis in Osr1 females is confirmed by comparing total area of steatosis with the WT female (p<0.01) (Fig 2D).

### Osr1 heterozygous mice display hepatic overexpression of key modulators of lipogenesis

**Fig 3:**
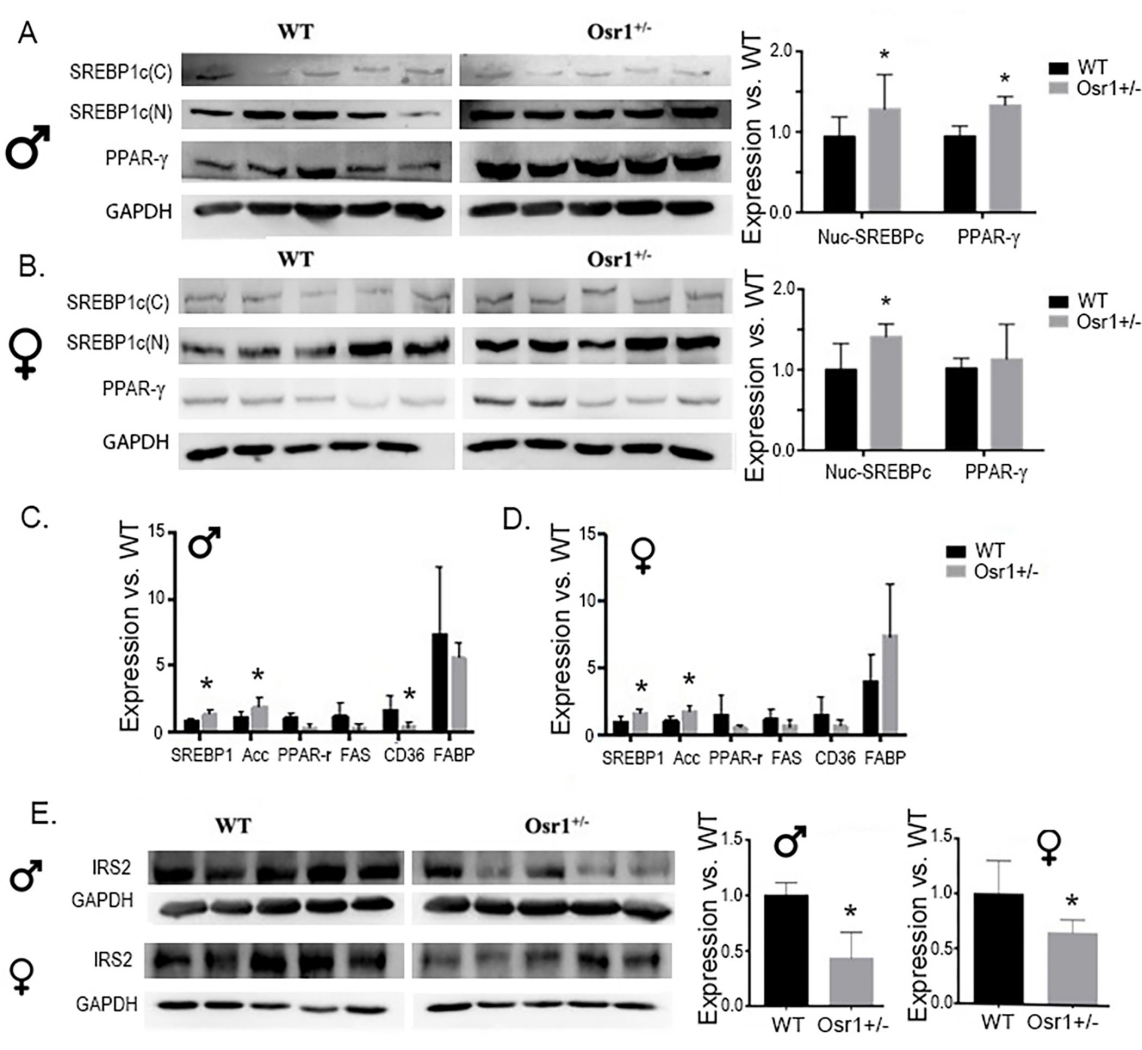
Osr1 heterozygous mice display hepatic overexpression of key modulators of lipogenesis. (A). The nuclear SREBP1c and PPAR-γ were significantly increased in male Osr1 mice compared to WT. (B). SREBP1 was significantly enhanced in Osr1 heterozygous female mice. (C). *SREBP1* and *ACC* was significantly increased in *Osr1* heterozygous male mice compared to wildtype. *CD36* was significantly increased in WT mice. (D). *SREBP1* and *ACC* was significantly increased in *Osr1* heterozygous female mice compared to wildtype. (E). Both *Osr1*^+/-^ male and female mice exhibited significantly impaired IRS-2 expression compared to WT mice. Data is presented as Mean±SD, N = 5; *p<0.05.

Hallmark characteristics of NAFLD phenotype are increased fatty acid concentration and lipogenesis in the liver. Protein expression for SREBP1, PPAR-γ and IRS-2 in the liver was measured by Western Blot analysis. Osr1 males exhibited significantly greater expression of both nuclear SREBP1 and PPAR-γ (p<0.05) (Fig 3A). Unlike male Osr1 mice, only nuclear SREBP1, but not PPAR-γ expression was significantly increased in female Osr1 mice versus the WT group (Fig 3B). These results encouraged exploration into expression of lipogenesis genes including *Srebp1*, *Acc*, *Ppar-γ*, *Fas*, *Cd36*, and *Fib*. Both the male and female Osr1 mice displayed higher expression of *Srebp1* and *Acc* in the liver, compared to WT liver. The male Osr1 mice also presented decreased expression of *Cd36* (Fig 3C and 3D). Compared to WT males, both the *Osr1* males and females exhibited significantly diminished expression of IRS-2 (Fig 3E, *p*<0.05).

### Osr1 heterozygous mice experienced decreased bile acid synthesis in the liver

**Fig 4:**
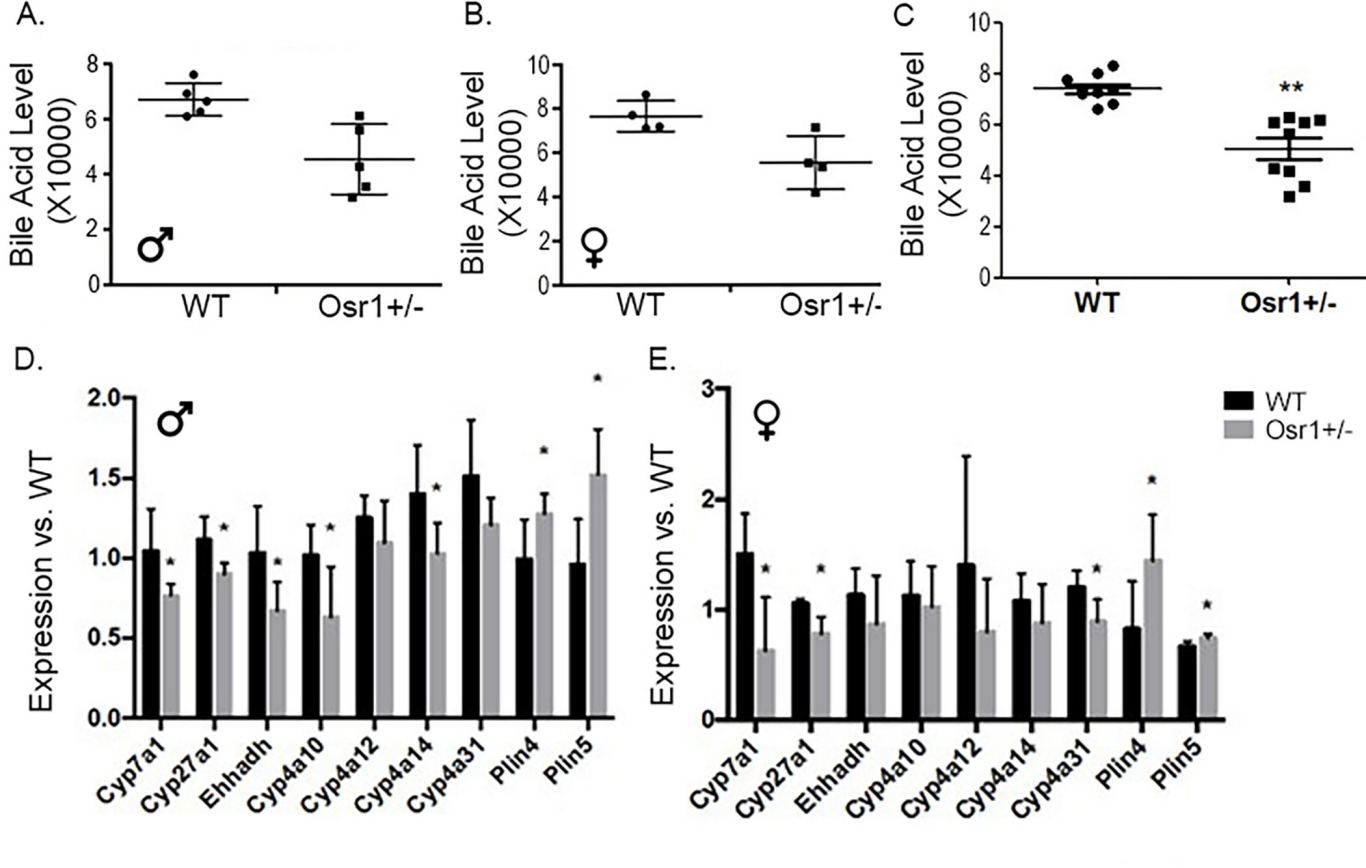
Osr1 heterozygous mice experienced decreased bile acid synthesis in the liver. (A). Osr1 male mice had a decreased trend of hepatic bile acid compared to WT mice. (B). Osr1 female mice had a decreased trend of hepatic bile acid compared to WT mice. (C). Combined results of Osr1 male and female mice show a decreased trend of hepatic bile acid compared to WT mice. (D) Osr1 heterozygous male mice had significantly decreased expression of *Cyp7a1*, *Cyp27a1*, *Ehhadh*, *Cyp4a10* and *Cyp4a14* compared to WT male mice. *Plin4* and *Plin5* were significantly increased in Osr1 male mice. (E). *Cyp7a1*, *Cyp27a1*, *Ehhadh*, and *Cyp4a31* were significantly reduced in Osr1 knockdown female mice. Osr1 female mice had significantly increased gene expression of *Plin4* and *Plin5*. Data is presented as Mean±SD, N = 5; *p<0.05.

Total bile acid from hepatic tissue was extracted and quantified to assess bile acids synthesis. An observed trend of decreased bile acid synthesis in both Osr1 male and female mice were noticed although the difference was not significant (Fig 4A and 4B). Since bile acid levels do not appear to be sex-dependent, we combined those of the male and female mice and the difference of bile acid between WT and Osr1 groups was reanalyzed. The results showed a significantly decreased total bile acid in Osr1 mice (p<0.05) compared to the WT mice (Fig 4C).

RT-PCR analysis was performed for bile acid synthesis genes involved in the classic and alternative pathways including *Cyp7a1*, *Cyp27a1*, *Ehhadh*, *Cyp4a10*, *Cyp4a12*, *Cyp4a14* and *Cyp4a31*. Osr1 male mice had significantly decreased expression for *Cyp7a1*, *Cyp27a1*, *Ehhadh*, *Cyp4a10*, and *Cyp4a14* (p<0.05) (Fig 4D). Osr1 heterozygous female mice experienced a statistically significant downregulation (p<0.05) of *Cyp7a1*, *Cyp27a1*, and *Cyp4a31* (Fig 4D).

In addition to bile acid synthesis genes, we assessed two known lipid droplet targeting proteins that regulate accumulation of lipid droplets in response to high lipid oxidative metabolism. *Perilipin 4 (Plin4)* and *perilipin 5 (Plin5)* protect lipid droplet storage against lipases and encourage lipid collection. Targeted overexpression of these genes can cause steatosis. In both male and female Osr1 mice, *Plin4* and *Plin5* were significantly increased compared to WT (p<0.01 for male and p<0.05 for female) (Fig 4E).

### Osr1 heterozygous mice displayed overactivation of Akt/mTOR signaling in the liver

**Fig 5:**
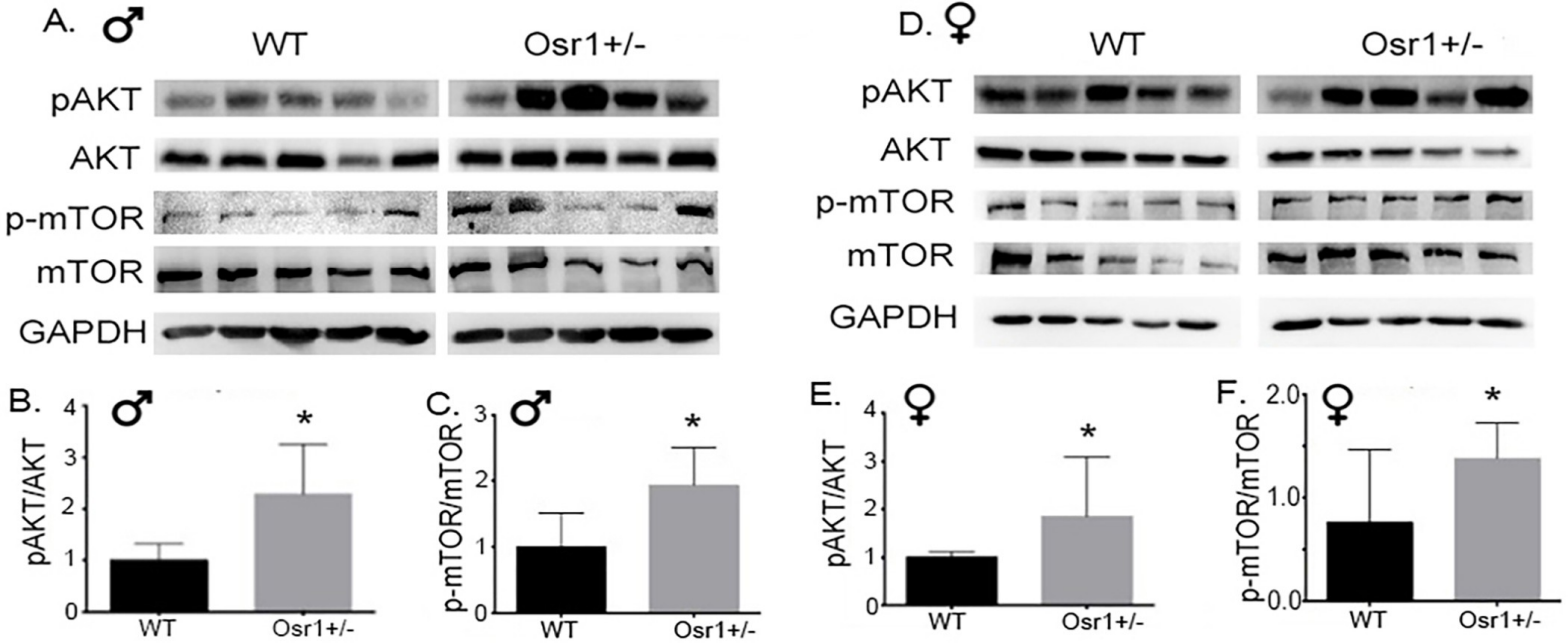
Osr1 heterozygous mice overactivated AKT and mTOR signaling in liver. (A). Immunoblot of hepatic Akt, p-AKT, mTOR and p-mTOR in male mice. (B) Osr1 heterozygous male mice had elevated p-AKT/AKT compared to WT male mice. (B). Osr1 heterozygous male mice had elevated p-mTOR/mTOR compared to WT male mice. (C). Immunoblot of hepatic Akt, p-AKT, mTOR and p-mTOR in female mice. (D). Osr1 heterozygous female mice had elevated p-AKT/AKT compared to WT female mice. (E). Osr1 heterozygous female mice had elevated p-mTOR/mTOR compared to WT female mice. Data is presented as Mean±SD, N = 5; *p<0.05.

Assessing immunoblot results, we detected the activation of Akt/mTOR signaling in both Osr1 males and females. The amount of p-Akt and p-Akt/Akt was upregulated significantly in both the Osr1 males and females (Fig 5A, 5B, 5D and 5E). With respect to mTOR and p-mTOR, both the male and female Osr1 mice displayed enhanced levels of p-mTOR and p-mTOR/mTOR (Fig 5A, 5C, 5D and 5E).

### Osr1 heterozygous mice exhibited more liver inflammation in hepatic tissue

**Fig 6:**
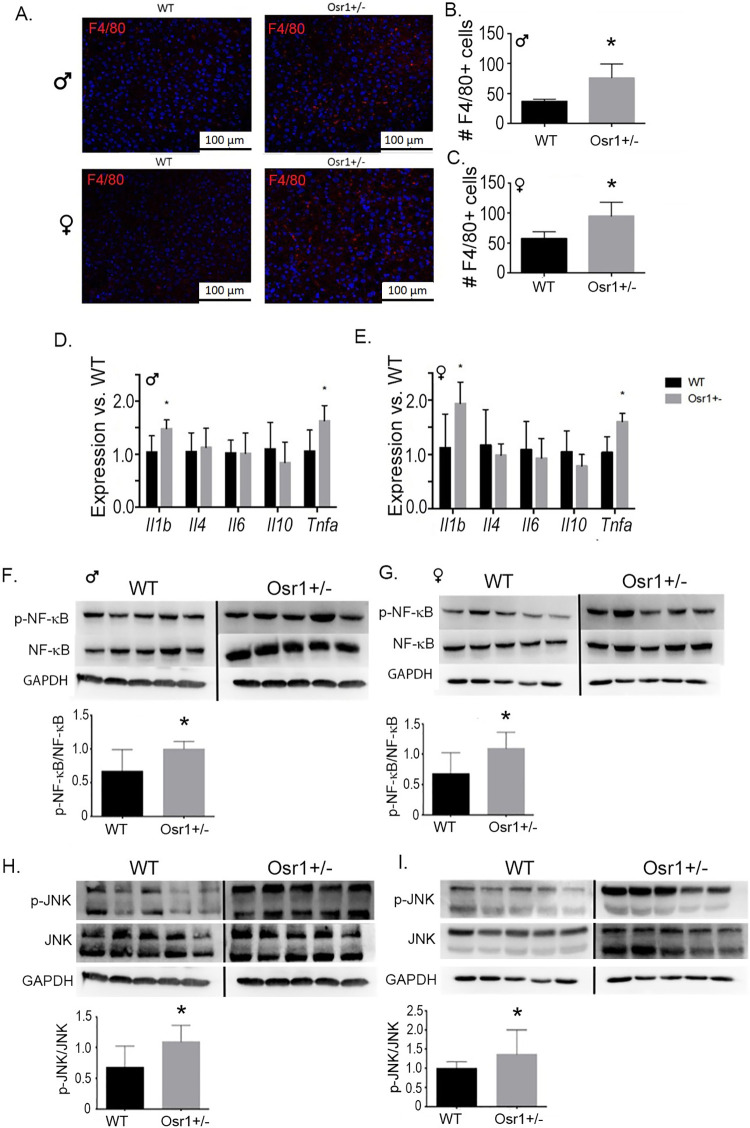
Osr1 heterozygous mice exhibited more liver inflammation in hepatic tissue. (A). F4/80 Immunofluorescence results for macrophages in hepatic tissue. Scale = 100 μm. (B). Male mice F4/80 positive cell quantification. (C). F4/80 positive cell quantification in female mice. (D). Osr1 male mice exhibited significantly upregulated pro-inflammatory *Il-1β* and *TNF-α*. (E). Osr1 female mice experienced significant upregulation of inflammokines *Il-1β* and *TNF-α*. (F). Osr1male mice exhibited significantly upregulated pNF-κB/NF-κB compared to male WT mice. (G). Osr1 female mice experienced significant upregulation of pNF-κB/NF-κB. (H). pJNK/JNK was significantly increased in Osr1 heterozygous males. (I). Osr1 heterozygous females displayed significant upregulation of pJNK/JNK signaling compared to WT female mice.

We performed an immunofluorescence assay using F4/80 antibody to detect the presence of macrophages in the liver. Both the Osr1 male and female mice had significantly increased infiltration of macrophages compared to the WT group (p<0.05 for male and p<0.01 for female) (Fig 6A–6C). We further assessed inflammatory activity by evaluating the gene expression of *Il-1β*, *Il-4*, *Il-6*, *Il-10*, and *TNF-α*. Both the male and female Osr1 mice had increased expression of *Il-1β* and *TNF-α* in the liver (Fig 6D and 6E), which coincides with the elevated number of macrophages observed.

JNK and NF-κB signaling pathways are activated by phosphorylation, which is facilitated largely by pro-inflammatory cytokines Il-1β and TNF-α. Osr1 males experienced significantly higher levels of p-NF-κB, NF-κB, as well as p-NF-κB/NF-κB compared to WT males (p<0.05) (Fig 6F). Osr1 females likewise expressed significantly greater levels of p-NF-κB and p-NF-κB/NF-κB (p<0.05), but not NF-κB (Fig 6G). For JNK signaling, both the male and female Osr1 displayed a statistically significant increase of pJNK and pJNK/JNK ratio (p<0.05) (Fig 6H). In female Osr1 mice, there was also a higher level of JNK observed in the liver (p<0.05) (Fig 6I).

## Discussion

Previous findings from our lab have explored the role of Osr1 to inhibit hepatic injury caused by HFD and DEN, potentially serving to depress its progression to HCC.(20) We report in this pre-cancer model that Osr1 heterozygote mice exhibited more steatosis. Thus, we hypothesize that disrupting Osr1 expression breaks the lipid homeostasis and induces inflammation in the liver, further promoting the progression of NAFLD. To test this hypothesis, the current study employed a mouse NAFLD model induced by HFD for 10 weeks. Knocking down Osr1 in mice with HFD-induced obesogenic conditions led to advanced hepatic steatosis compared to WT mice, independent of sex. This phenotype is associated with increased lipogenesis, diminished bile acid synthesis, and increased hepatic inflammation. We further identified that activation of several key signaling pathways, including Akt/mTOR signaling, JNK, and NF-κB signaling were associated with disrupted Osr1 expression in the liver. Based on our results, the Osr1 gene appears to play a hepatoprotective role in the progression of NAFLD.

In our study, sex as a biological variance is fully evaluated to determine if the important role of Osr1 on NAFLD progression is sex-dependent. The liver is a sexually dimorphic organ, exhibiting differences in steroid metabolism, number of parenchymal and nonparenchymal cells, and varying interactions between immune and endocrine systems. NAFLD pathogenesis is closely associated with dysmetabolic features [21]. In animal and human studies, the female sex is protected from dysmetabolism [22]. In our study, liver histology did reveal that female mice experienced less severe steatosis than males, regardless different genotypes. However, knocking down Osr1 similarly caused more severe hepatic steatosis than WT mice regardless of male or female sex. Consistently, overall changes in the gene expression and the signaling pathways in Osr1 mice versus the WT mice were not different by sex, although expression change of individual genes or proteins such as the PPARγ was not same. Likely the minor difference is a reflection of the sex variance in lipid metabolism. Nonetheless, our results suggest that the role of Osr1 in lipid metabolism and liver inflammation is not sex-dependent.

Worsened hepatic steatosis is a major pathological change in the *Osr1*^*+/-*^ liver. It is noted that the body weight between the *Osr1*^*+/-*^ and *WT* mice was not statistically different upon 10-week HFD. This fact suggests to us that the role of Osr1 in lipid metabolism is not obese related, highlighting an important role of Osr1 in hepatic lipid metabolism in liver. The enhanced expression of lipogenesis genes and associated overactivation of Akt/mTOR signaling indicated dysfunctional lipogenesis and hepatic lipid metabolism. Osr1 knockdown mice exhibited enhanced expression of SREBP1 and ACC, indicative of increased hepatic fatty acid uptake and lipogenesis. Expression levels occurring in these groups coincide with expected results based on severe microvesicular and macrovesicular steatosis. In the liver, PPAR-γ mediates lipid metabolism, specifically targeting genes involved in *de novo* lipogenesis and free fatty acid import [23]. Akt/mTOR signaling regulates various hepatic, metabolic, and physiological processes including glucose and lipid metabolism [24]. This pathway promotes lipogenesis by positive regulation of lipogenic genes including the SREBP family of transcription factors responsible for fatty acid and cholesterol synthesis [25]. Akt/mTOR signaling was significantly enhanced in Osr1 mice, likely contributing to upregulation of *Srebp1*. Cholesterol and bile acid homeostasis plays a central role in metabolic health. Bile acid synthesis is a major pathway for hepatic cholesterol catabolism, via Cyp7a1 regulated classic pathway and Cyp27a1 regulated alternative (acidic) pathway [26]. A tightly regulated feedback mechanism is required in maintaining the lipid homeostasis. Dysregulation of this feedback signaling network significantly contributes to pathologies of NAFLD [27, 28]. The Osr1 mice presented lower amounts of bile acid in the liver associated with decreased expression of *Cyp7a1* and *Cyp27a1*, suggesting an important role of Osr1 in bile acid synthesis. It is possible that reduced bile acid synthesis results in a “cholesterol trap” in the liver, which further contributes to the development of cholestasis. To test this hypothesized mechanism, future study will need to focus on the status of hepatic cholesterol homeostasis in the Osr1 heterozygote mice exposed to HFD. Interestingly, it has been reported that disrupting *Cyp7a1* expression in the liver could induce hepatic inflammation [29], which is consistent with our observations. Nonetheless, our data suggested that Osr1 plays a role in regulating lipid metabolism in the liver.

Macrophage infiltration and classical activation of proinflammatory macrophages by inflammatory cytokines IL-1β and TNF-α are powerful indicators of inflammation in hepatic tissue [30]. The two classical signaling pathways associated with hepatic inflammation involve mitogen activated JNK and NF-κB [31]. Increased JNK and NF-κB are the major source of damaging inflammatory cytokines in NASH and apoptosis. These factors are distinguished by the production of IL-6, IL-10, and TNF-α [32]. In our study, Osr1 mice displayed more macrophage infiltration, higher hepatic expression of *Il-1β* and *Tnfα*, together with overactivation of JNK and NF-κB signaling. These data support Osr1 as a critical mediator for liver inflammation in NAFLD progression. To be noted, Osr1 has been found to be strongly expressed in macrophages, whose infiltration into the liver is more profound in Osr1 heterozygous liver [20].

Both the classic “two hit” hypothesis and the “multiple parallel hit” model have highlighted lipotoxicity-induced oxidative stress, endoplasmic reticulum (ER) stress and immunological responses involving macrophages to be the central drivers of hepatic injury in NAFLD/NASH [5, 33, 34]. In the current study, our results could not reveal if increased liver inflammation in the *Osr1*^*+/-*^ mice is a cause or a consequence of the steatosis, thus it is unclear if the worsened steatosis or the enhanced hepatic inflammation is the driving force for NAFLD progression. To solve this question, future study needs to identify the direct targets of Osr1 in liver. In addition, mouse models with Osr1 disruption or deletion specifically in hepatocytes or macrophages should be utilized to understand the important role of Osr1 in lipid metabolism and hepatic inflammation during NAFLD progression.

In summary, our study provides evidence that Osr1 plays an important role in regulating the lipid homeostasis and hepatic inflammation, whose disruptions contribute to NAFLD progression.

## Supporting information

S1 Raw images (PDF)
