# Supplementary figures and images for: Disrupting Osr1 expression promoted hepatic steatosis and inflammation induced by high-fat diet in the mouse model
Source: PLoS One. 2022 Jun 3;17(6):e0268344. doi: 10.1371/journal.pone.0268344 (PMC9165803; doi:10.1371/journal.pone.0268344)

**Fig. 1 Male**

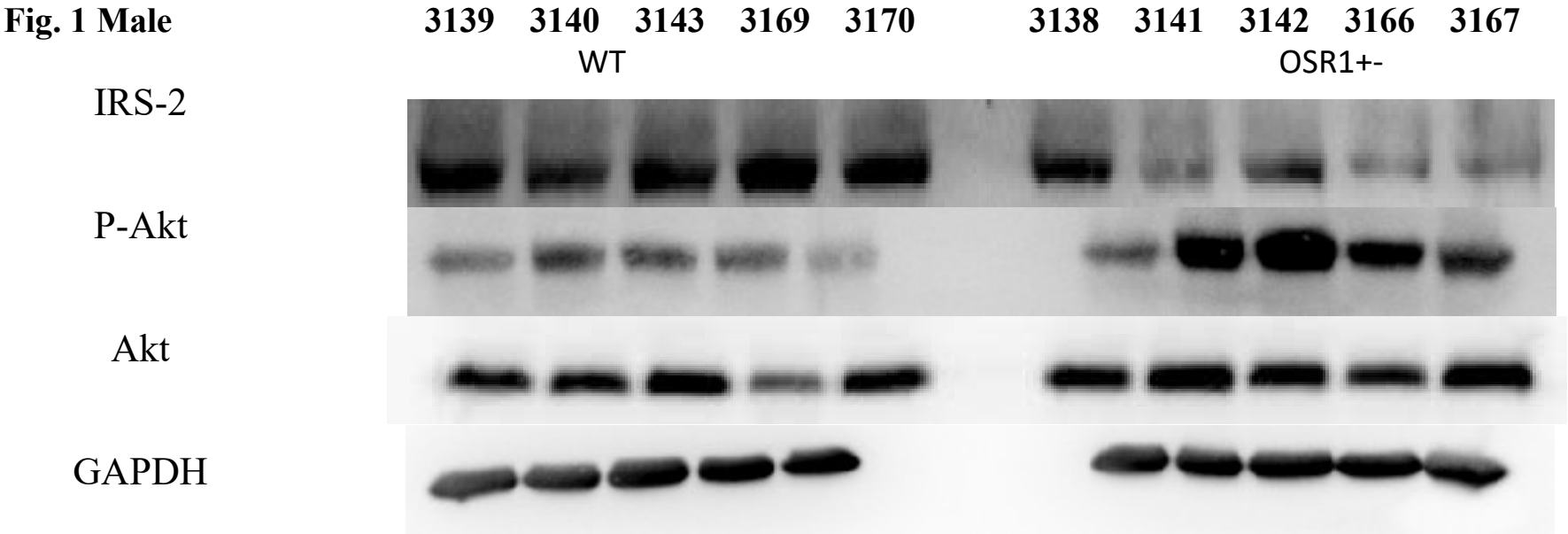

**Fig. 2 Male**

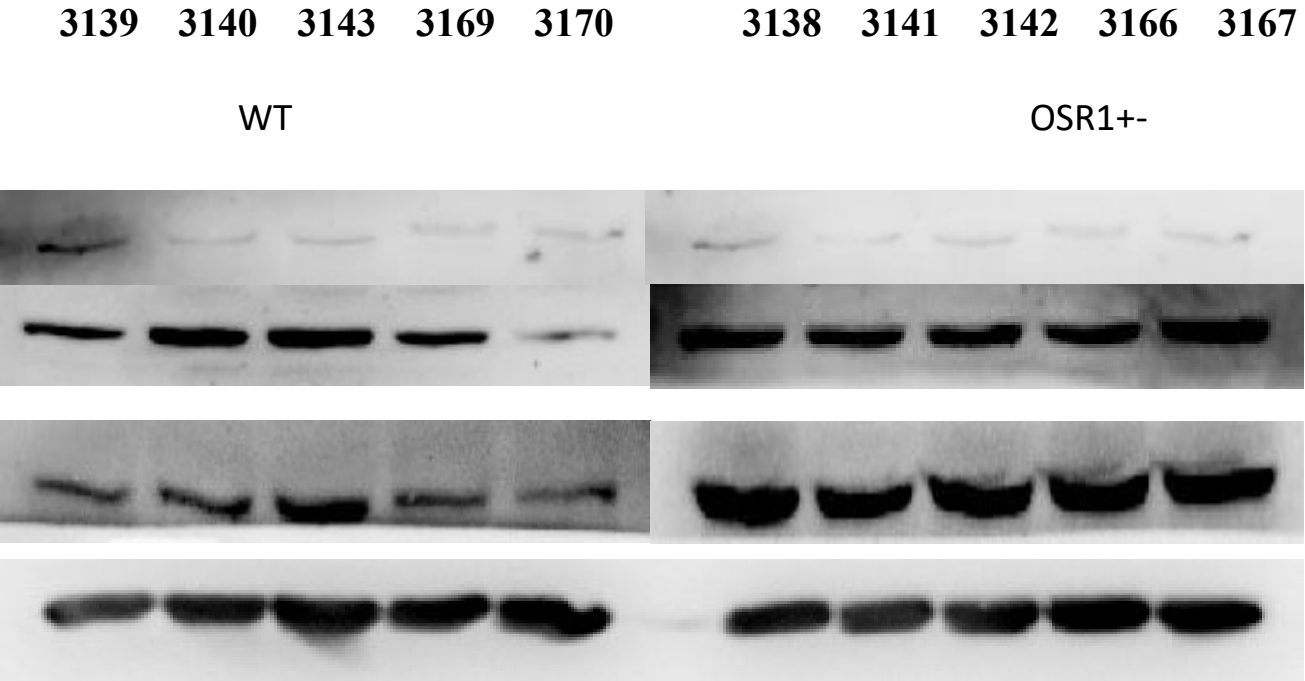

**Fig. 3 Male**

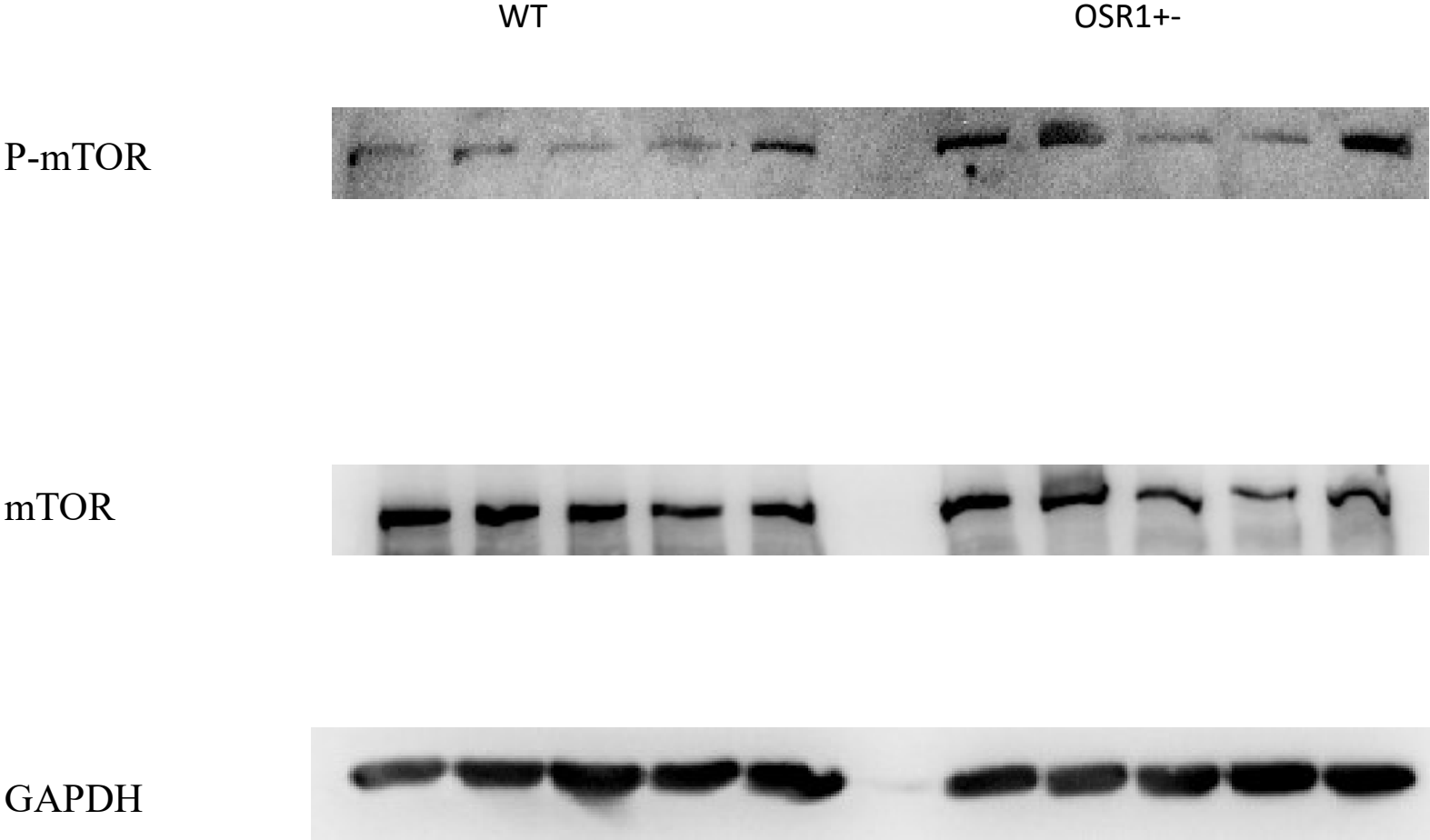

**Fig. 4 Male**

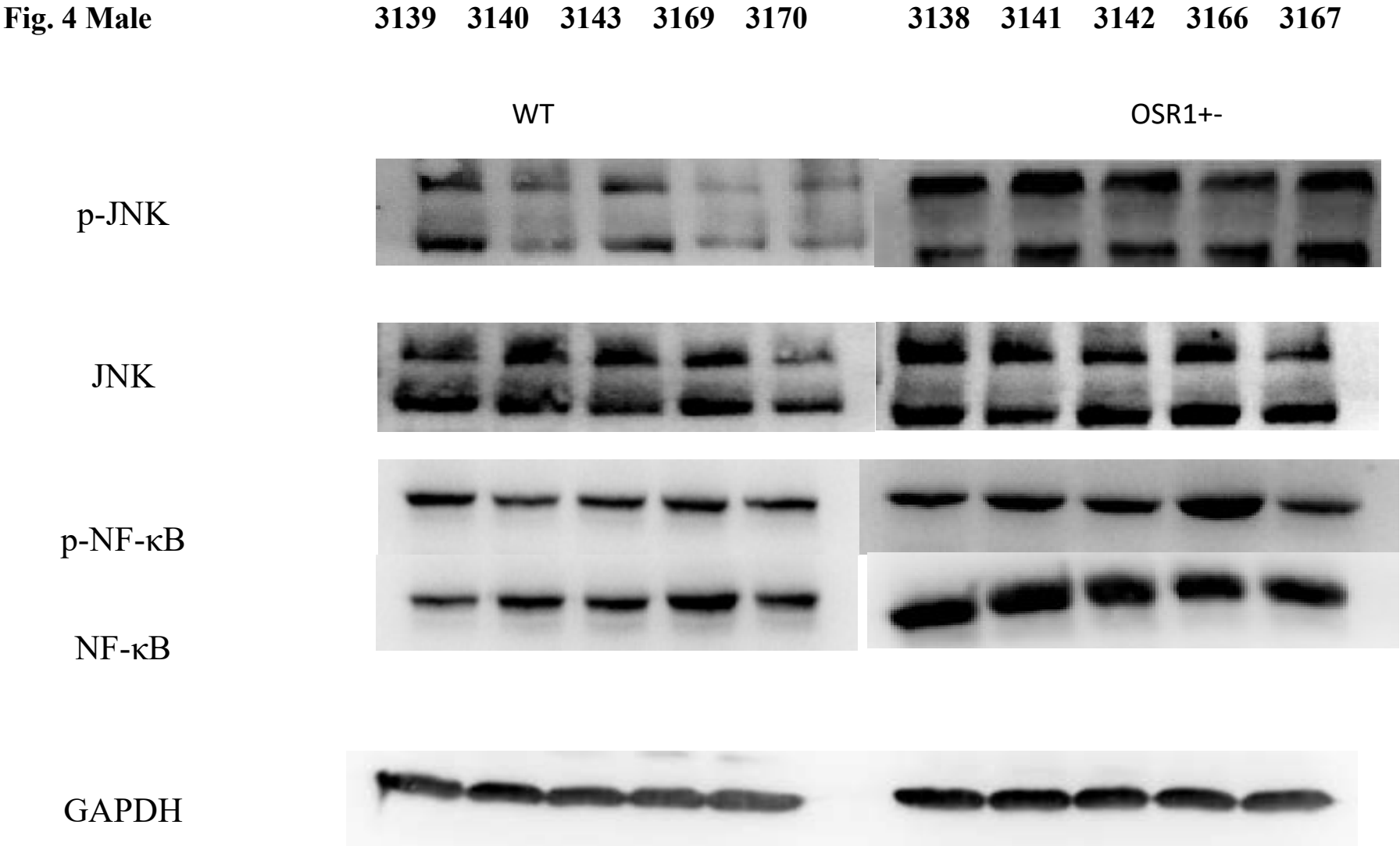

**Fig. 1 Female**

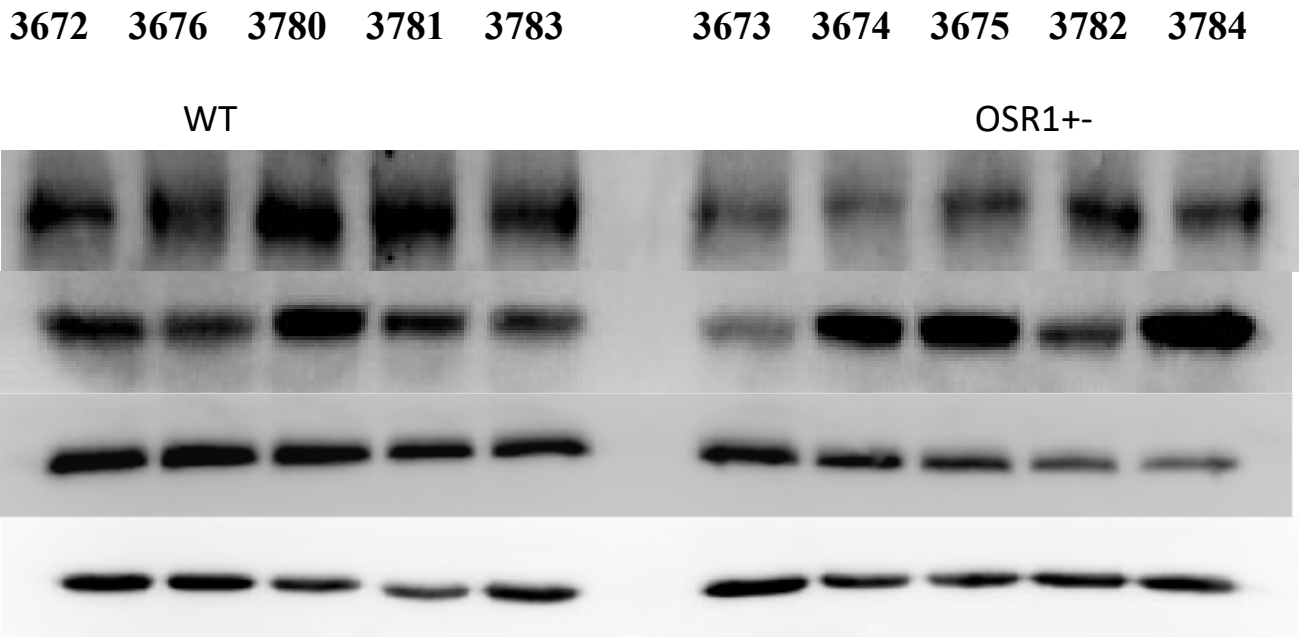

**Fig. 2 Female**

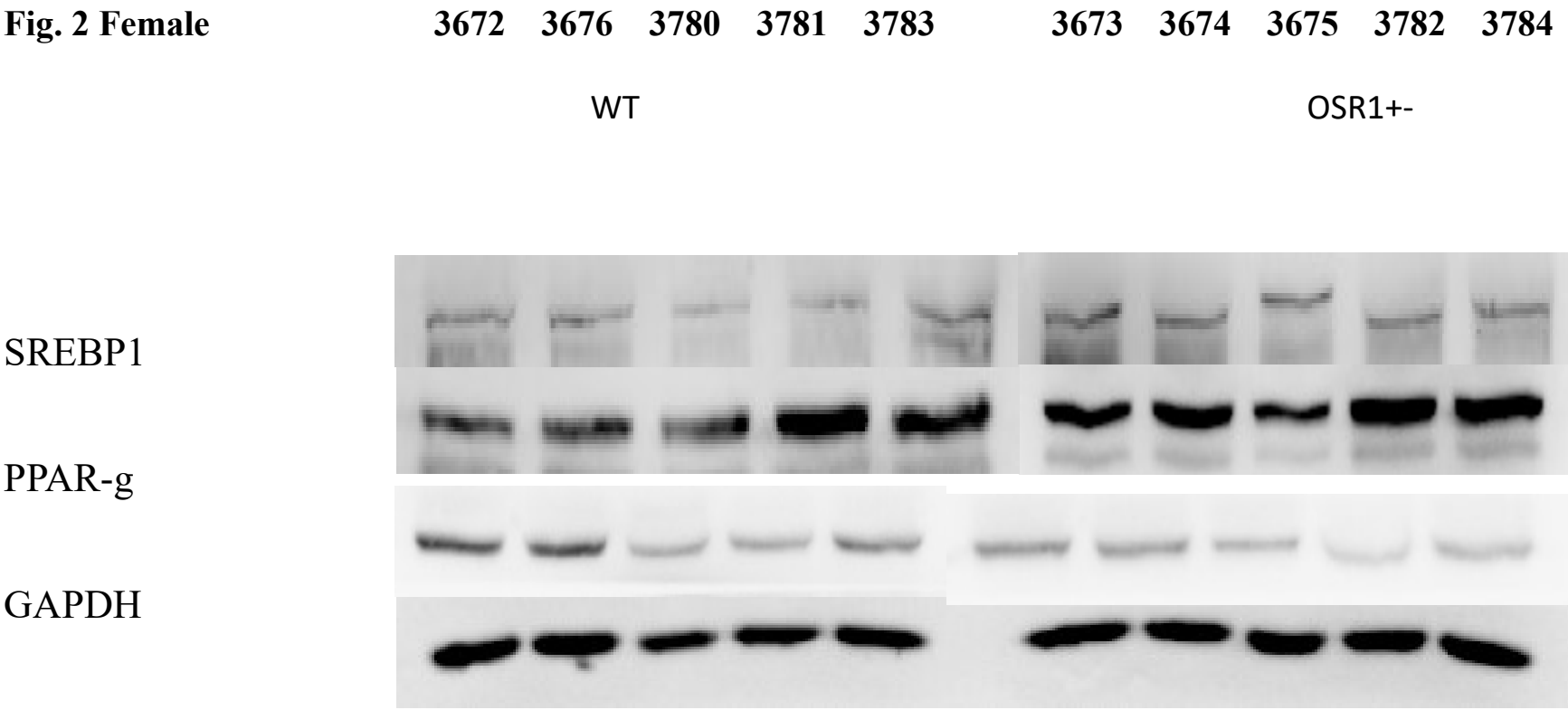

**Fig. 3 Female**

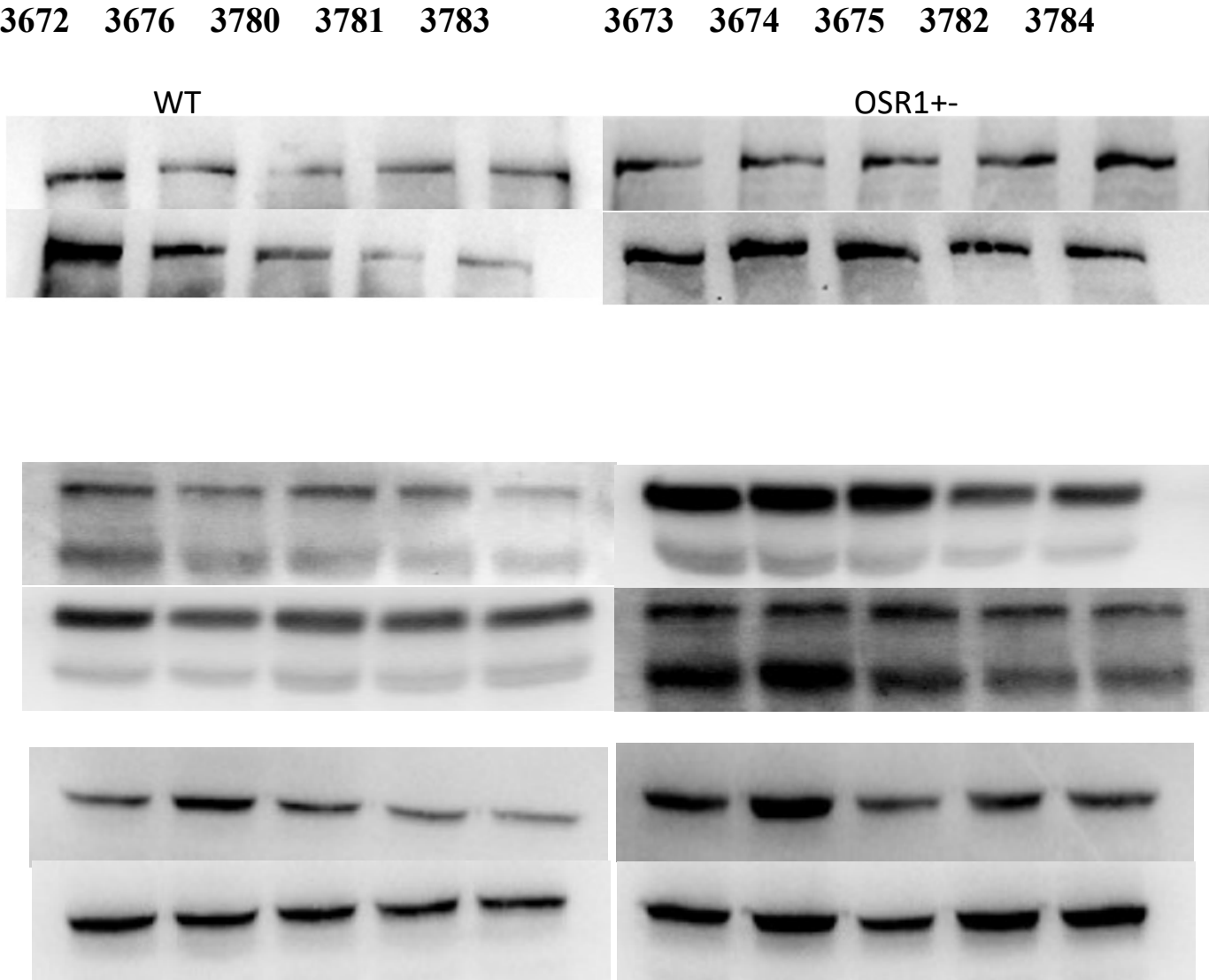

Supplement: S1 Raw images — (PDF) [file pone.0268344.s001.pdf]
